# Susceptibility to cognitive distortions: the role of eating pathology

**DOI:** 10.1186/s40337-015-0068-9

**Published:** 2015-09-04

**Authors:** Jennifer S. Coelho, Catherine Ouellet-Courtois, Christine Purdon, Howard Steiger

**Affiliations:** Eating Disorders Program, Douglas Mental Health University Institute, Montreal, Canada; Department of Psychiatry, McGill University, Montreal, Canada; Department of Psychology, Simon Fraser University, Burnaby, Canada; Department of Psychology, University of Waterloo, Waterloo, Canada

**Keywords:** Thought-shape fusion, Thought-action fusion, Cognitive distortion, Eating disorders

## Abstract

**Background:**

Thought-Shape Fusion (TSF) and Thought-Action Fusion (TAF) are cognitive distortions that are associated with eating and obsessional pathology respectively. Both involve the underlying belief that mere thoughts and mental images can lead to negative outcomes. TSF involves the belief that food-related thoughts lead to weight gain, body dissatisfaction, and perceptions of moral wrong-doing. TAF is more general, and involves the belief that merely thinking about a negative event (e.g., a loved one getting into a car accident) can make this event more likely to happen, and leads to perceptions of moral wrong-doing. However, the shared susceptibility across related cognitive distortions—TAF and TSF—has not yet been studied.

**Method:**

The effects of TSF and TAF inductions in women with an eating disorder (*n* = 21) and a group of healthy control women with no history of an eating disorder (*n* = 23) were measured. A repeated-measures design was employed, with all participants exposed to a TSF, TAF and neutral induction during three separate experimental sessions. Participants’ cognitive and behavioral responses were assessed.

**Results:**

Individuals with eating disorders were more susceptible to TSF and TAF than were control participants, demonstrating more neutralization behavior after TSF and TAF inductions (i.e., actions to try to reduce the negative effects of the induction), and reporting higher levels of trait TAF and TSF than did controls.

**Conclusions:**

Individuals with eating disorders are particularly susceptible to both TAF and TSF. Clinical implications of these findings will be discussed.

## Background

According to cognitive-behavioral theories of obsessive-compulsive disorder (OCD), catastrophic misinterpretations of the meaning of intrusive thoughts contribute to obsessive symptomatology (e.g., [1]). Thought-action fusion (TAF) is a cognitive distortion involving the belief that thoughts about a negative event (e.g., a loved one getting into a car accident) make this event more likely to happen, and that thoughts and actions are morally equivalent [2]. Metacognitive beliefs, such as TAF, have been proposed to play a role in the maintenance of OCD symptoms [3]. There has been a growing body of literature over the past two decades on the relationship between TAF and obsessive-compulsive pathology, with some researchers proposing that TAF may play a causal role in the development of obsessional intrusive thoughts [4]. However, TAF does not appear to be specific to OCD. Recent research suggests that there are no differences in TAF scores between individuals with OCD and those with other anxiety diagnoses (i.e., panic disorder, social anxiety, and generalized anxiety disorder) [5]. In line with the potential generalizability of TAF across psychopathologies, Shafran and Rachman [1] note that TAF may also be prominent in those with anorexia nervosa.

Although general obsessional beliefs have been investigated in individuals with eating disorders (e.g., [6]), there has been relatively little research into the phenomenon of TAF in those with eating disorders. High TAF scores have been reported in a sample of individuals with anorexia nervosa (*M* = 39.2) [7], as well as a transdiagnostic sample of individuals with eating disorders (*M* = 36.48) [8]. The TAF scores of participants with eating disorders exceed the mean total TAF scores of individuals with OCD (e.g., *M* = 31.5) [9]. However, in a study that directly compared total TAF scores in those with eating disorders, OCD, and healthy controls, no significant main effects of group were found [10]. In this experimental study conducted by Coelho and colleagues [10], an induction was used prior to measuring TAF scores, and those with OCD who received a neutral induction (thinking about the sensory aspects of being in a park) reported lower TAF scores than did other groups. It is possible that the experimental induction temporarily reduced TAF susceptibility in those with OCD, given that this induction was found to decrease negative affect in participants with OCD. The relatively low levels of TAF in those with OCD in the previous study may be accounted for by the fact that negative affect (i.e., anxiety and depression) mediates the association between TAF and OCD [11]. Therefore, further research aimed at addressing the potentially confounding factor of negative affect is needed to further assess differences in TAF susceptibility across eating disorders and OCD. Generally, research demonstrates that individuals with eating disorders are particularly susceptible to obsessional thinking styles and cognitive distortions.

Shafran and colleagues [2], who conducted the first experimental investigation of TAF, postulated that a variant of TAF is present in those with eating disorders. This phenomenon, which they called thought-shape fusion (TSF), was proposed to involve the belief that merely thinking about consumption of a high-caloric food leads to weight gain and body dissatisfaction [12]. Like TAF, TSF also was thought to involve the confounding of actions and thoughts, such that merely thinking about eating forbidden foods leads to perceptions of moral wrong-doing. Numerous studies have supported the link between TSF and eating pathology, with individuals with eating disorders reporting higher levels of TSF than healthy controls (e.g., [8, 10, 13]). TSF can be induced by asking individuals to imagine consuming a high-caloric food and writing down a sentence about eating the imagined food. TSF inductions lead to higher levels of anxiety, guilt, perceptions of weight gain, body dissatisfaction, and moral wrong-doing, as well as neutralizing behavior to try to reduce the effects of the induction, such as crossing out the sentence about eating (e.g., [8]).

Researchers have assessed general vulnerability to TSF using a trait measure [14], which taps into general beliefs about the effects of thinking about food on weight gain, perceptions of fatness, and moral-wrong doing. State vulnerability to TSF after inductions has been assessed using a state TSF measure that assesses the core components of the TSF experience (i.e., ratings of current anxiety, guilt, moral wrong-doing, perceptions of weight gain, body dissatisfaction, and urges to engage in neutralizing behavior). Trait and state TSF appear to be distinct, such that state TSF levels are higher in individuals who have been asked to imagine eating a high-caloric food than those who have been asked to imagine a neutral situation (and trait TSF levels across these groups do not differ) [15]. Therefore, state TSF appears to measure current emotional reactions, whereas trait TSF measures a general propensity towards the belief about negative effects of mere thoughts about food.

TSF inductions have been well-established to increase state TSF, particularly in individuals with eating disorders (e.g., [10, 16]). TSF inductions have also been demonstrated to increase state TSF in healthy control women who are in the normal weight range [17]. However, some studies have demonstrated that trait TSF scores are influenced by TSF inductions (e.g., [10, 17]). The studies demonstrating a significant effect of inductions on trait TSF were limited by the lack of a baseline measure of trait TSF prior to exposure to an induction. Recent research employing a within-subject design has demonstrated stability in trait TSF across time [18]. However, the distinct nature of the state and trait TSF measures requires further exploration.

Although TSF susceptibility in individuals with eating disorders has been established using a TSF induction, TAF inductions have not yet been investigated in individuals with eating disorders. Researchers have induced TAF in healthy control participants by asking them to imagine adverse events, such as a loved one having a car accident. TAF inductions have been demonstrated to provoke anxiety, perceived likelihood of the imagined event occurring, and perceived moral wrong-doing in non-clinical samples [19]. Given the susceptibility of individuals with eating disorders to both TSF and TAF, we set out to explore whether TAF could be experimentally induced in individuals with eating disorders, just as TSF has been induced. We used a repeated measures design in which two groups of participants (healthy controls and those with eating disorders) were exposed to a neutral, TSF and TAF induction in three separate experimental sessions. The following predictions were made:

## Hypothesis 1. Individuals with eating disorders will be more susceptible to both TSF and TAF inductions than will healthy controls

We predicted that individuals with eating disorders would report significantly more negative emotional responses to TAF and TSF inductions, and engage in more neutralization behavior (e.g., actions to try to reduce the effects of the negative thoughts) than would healthy controls.

## Hypothesis 2. Individuals with eating disorders will report more general susceptibility to TAF and TSF than will healthy controls

We anticipated more general susceptibility to TSF and TAF in participants with eating disorders, and predicted that participants with eating disorders would report higher scores on the TAF scale and trait TSF measures than would healthy controls and that scores on these measures would be stable across inductions (therefore representing trait measures of these constructs, rather than reactivity to inductions of TAF or TSF).

## Method

### Participants

Women between the ages of 18–45 were recruited to participate, with recruitment targeting a sample of women with an eating disorder, as well as a healthy control sample. Recruitment was limited to women, given the high prevalence of females in eating disorder treatment settings. Clinical participants with a principal diagnosis of an eating disorder (in accordance with DSM-IV-TR criteria) [20] were recruited from a specialized treatment program for eating disorders. The diagnosis of participants with eating disorders was confirmed based on responses to the eating disorders module of the Structured Clinical Interview for DSM-IV Axis I Disorders– Research Version (SCID-I-RV) [21]. Non-clinical control participants were recruited through on-line advertisements in the local community.

Exclusion criteria for participants with eating disorders and non-clinical participants alike included a current or past diagnosis of obsessive-compulsive disorder, and current psychotic symptoms. Recruitment of participants with eating disorders was additionally limited to those with a minimum body mass index of 13, to limit cognitive deficits that may accompany extremely low weight. Additional exclusion criteria for control participants included a current or past history of an eating disorder, and current psychological treatment with a mental health professional.^1^ A total of 24 women with eating disorders were recruited; however, three individuals did not complete all sessions of the study. The final sample consisted of 21 women with an eating disorder, and 23 control women.

#### Measures

Participants were given the choice of completing measures in either English or French, given the bilingual nature of the area where this study was conducted. A total of 36 individuals completed the study in French, and 8 completed it in English.

#### Target thought priming ratings

A 3-item questionnaire was provided to assess the vividness of the imagined situation, and the extent to which individuals found it difficult and distressing to imagine this situation. This measure was developed for a previous study [22], and has been demonstrated to have adequate internal reliability in research on TSF inductions (e.g., [10]). All ratings were performed on a 100-mm VAS, with 0 = not at all and 100 = totally. This measure was used as a manipulation check, to assess vividness of the imagery across inductions. Scores on the two questions assessing the difficulty/distress associated with the imagery were averaged, to assess the dependent variable of negative emotional response associated with the inductions. The obtained Cronbach’s alpha for this composite measure during the neutral induction was .83.

#### State Thought-Shape Fusion Scale (State TSF Scale) [10]

Participants were asked to consider their thoughts and feelings in response to the induction (see Procedure), and to rate the following six items: anxiety, guilt, likelihood of weight gain, extent to which they felt fatter, the extent to which imagining the situation and/or writing the sentence was morally wrong, and the extent to which they wanted to reduce the effects of imagining the situation/writing the sentence. Participants rated these items on a VAS of 0–100 (0 = ‘not at all’, and 100 = ‘completely’). A total score on this measure is obtained by averaging answers across the 6 items. For the purposes of testing Hypothesis 1 (emotional reactions to TSF and TAF inductions), two subscales were created from this measure: an emotional/behavioral subscale (i.e., average of items assessing anxiety, guilt, perceived moral wrong-doing, and urges to neutralize), and a body-related subscale (i.e., average of the two items assessing perceived weight gain and fatness). These subscales have not yet been validated. The obtained Cronbach’s alpha for the emotional/behavioral subscale was acceptable (.68), and was good for the body-related subscale (.85).

#### Trait Thought Shape Fusion Scale – Short Version (TSF-trait scale) [14]

The TSF scale is a validated 14-item questionnaire assessing general tendencies to experience thought-shape fusion. Participants are asked to rate the extent to which they generally agree with a series of statements that tap into the construct of TSF, including body dissatisfaction (e.g., “I feel fatter after thinking about eating “fattening/forbidden” foods (e.g., chocolate)”, perceptions of weight gain (e.g., “Just picturing myself gaining weight can really make me gain weight”) and moral wrong-doing (e.g., “If I think about breaking my diet, it is almost as unacceptable as really breaking my diet”). Items were scored on a 5-point Likert Scale (0 = not at all; 4 = completely). Four additional questions have been added to the measure to include ratings of the frequency, impact, importance of suppression, and uncontrollability of TSF-related thoughts, using the same 5-point Likert Scale; however, this clinically-relevant subscale has not yet been fully validated as part of the TSF measure. Therefore, we calculated two separate scores for this measure—the total trait TSF score (based on the sum of scores on the original 14 item measure, Cronbach’s alpha = .96), and the clinically-relevant subscale (based on the sum of scores on the 4 additional items, Cronbach’s alpha = .95).

#### Thought Action Fusion Scale (TAF scale) [2, 23]

The TAF scale is a 19-item questionnaire, with higher total scores reflecting higher levels of TAF. The psychometric properties of this measure have been established, including scale reliability and validity, with a recent factor analysis supporting the use of a total TAF score [24]. The obtained Cronbach’s alpha for this measure after the neutral induction was .89.

### Procedure

Participants with eating disorders were recruited through the Eating Disorders Program at the Douglas Mental Health University Institute. Individuals were provided with information about the study, and those who expressed interest were scheduled for an in-person experimental session. Non-clinical participants were recruited through advertisements in the local community. Pre-screening of non-clinical participants who indicated interest in the study was done by telephone, and individuals who met inclusion criteria were scheduled for an in-person appointment. Upon arrival for the session, participants signed an informed consent. The experimenter then asked participants to imagine one of three situations (i.e., the neutral, TSF or TAF induction). A within-subjects design was employed, such that all participants were exposed to each of the three inductions. The order of inductions was random, with rotation through all 6 possible combinations across participant groups. The description of the inductions is below.

#### TSF induction

As in previous research [12], participants were asked to think about food(s) that they considered to be fattening or high in calories, and to imagine eating large quantities of this food. During this induction, participants were prompted to write down the sentence “I am eating ________” (inserting the name of the food(s) they imagined), and to focus on the sensory qualities of this act (i.e., the appearance, smell, and taste of the food) for 30 s.

#### TAF induction

An in vivo method of inducing TAF was used, as in previous research [19]. Participants were asked to identify a close friend or family member, and to write down the sentence “I hope _________ is in a car accident today”. Participants were then asked to focus on an image of this situation for 30 s.

#### Neutral induction

Following the neutral induction employed in previous research [10], participants were asked to think about being in a local park, and to imagine the sensory aspects of this situation (i.e., the sight, sounds, smells, and tactile aspects of being in the park). Participants were asked to write down the sentence “I am hearing __________” (inserting the name of one of the sounds that they imagined), and to focus on this image for 30 s.

Upon completing the induction, participants were asked to complete a series of questionnaires, including the TAF scale, State TSF questionnaire, Target Thought Priming Ratings, and the TSF-trait scale (presented in that order). Participants were then given an opportunity to try to neutralize, or reduce the effects of having imagined the situation and written the sentence, as in previous research [8]. Participants were prompted by the experimenter that if they felt inclined, they could cross out the sentence, or write down another sentence (as examples of neutralizing behavior). The experimenter took note of whether participants chose to neutralize. Participants then completed a series of questionnaires as part of a separate study.^2^ They also completed modules of the SCID-I-RV [21] with the experimenter that related to the inclusion/exclusion criteria (eating disorders, obsessive-compulsive disorder, and psychotic symptoms), and demographic information was collected (i.e., age, number of years of post-secondary education). Finally, the height and weight of controls was measured by the experimenter, while the height and weight of participants with eating disorders was obtained from their chart, given that they were being regularly weighed in treatment.

At the end of the first session, participants were scheduled for their second and third sessions (with at least one day between sessions). The average time to complete all three sessions was 13.1 days (SD = 7.8). At sessions two and three, participants completed the remaining inductions, followed by the State TSF questionnaire, the TAF scale, Target Thought Priming Ratings, and the TSF-trait scale. The experimenter took note of whether participants chose to neutralize after the inductions. Participants were provided with a small honorarium, and were verbally debriefed at the end of the third session. All procedures were approved by the research ethics committee at the Douglas Mental Health University Institute (REB # 12–27) and at Simon Fraser University (study number 2013 s0872).

### Data analysis

SPSS Statistics (Version 22) was employed for all analyses. To test Hypothesis 1, repeated measures analyses of variance was employed, with participant group (eating disorder versus control) entered as a between-subjects factor. The main dependent measures for Hypothesis 1 were the scores on the difficulty/distress composite, and scores on the state TSF measure (body-related and emotional/behavioral subscales). Sample size analysis based on this main dependent measure was performed, which suggested that a sample of 44 participants would be sufficient to detect a medium-size effect for the planned analyses. A chi-square analysis was performed to assess differences in neutralization behavior across groups and inductions. Hypothesis 2 was also tested using repeated measures analyses of variances, with group entered as a between-subjects factor, and trait TSF scores and TAF scores as dependent variables (in two separate analyses). Assumptions of ANOVA, including normality, were verified prior to proceeding with analyses. An alpha of *p* < .05 was employed to assess statistical significance, with pairwise comparisons (corrected with a Bonferroni correction) employed where appropriate to break down significant interactions.

## Results

ANOVA demonstrated that participants with eating disorders had a lower mean BMI than did healthy controls, *t*(42) = 2.57, *p* ≤ .05 (*M* = 20.8, *SD* = 4.2 for those with eating disorders and *M* = 24.0, *SD* = 3.9 for controls). Group differences in mean number of years of post-secondary education (*t* (42) = 1.69, *p* = .09) and age (*t* (28.5) = .89, *p* = .38) did not reach statistical significance.

### Manipulation check: target thought priming ratings

A repeated measures ANOVA was performed on participants’ Target Thought Priming Ratings, with vividness scores across the three inductions entered as the dependent variable, and group as the between-subjects variable. There were significant differences in vividness ratings across inductions. Pairwise comparisons demonstrated that the mean vividness of the TAF induction was significantly lower than the vividness of the TSF or control inductions, *p* < .05 (see Table 1 for means). The interaction between group and induction did not reach significance.

**Table 1:** Mean Scores on the Target Thought Priming Ratings Scale across Inductions (Neutral, Thought-Shape Fusion (TSF), and Thought-Action Fusion (TAF)

		Neutral	TSF	TAF	ANOVA
Vividness Ratings		69.61 (20.97)^a^	67.81 (22.86)^a^	54.84 (25.70)^b^	Main effect^c^ of induction: *F*_2,84_ = 8.41, *p* < .001, *η*_*p*_^*2*^ = .167
					Induction group interaction: *F*_2,84_ = 2.31, *p* = .10, *η*_*p*_^*2*^ = .052
Difficulty/Distress Composite	Controls	9.09 (10.28)^a^	11.63 (13.65)^a^	75.41 (16.10)^b^	Main effect^c^ of induction: *F*_2,84_ = 134.12, *p* < .001, *η*_*p*_^*2*^ = .762
	Eating Disorders	19.59 (28.29)^a^	55.47 (32.08)^b^	77.76 (23.73)^c^	
					Induction^c^ group interaction: *F*_2,84_ = 15.92, *p* < .001, *η*_*p*_^*2*^ = .275

^a^Vividness ratings: significance set at p < .05

^b^Difficulty/Distress Composite: significance set a p < .0167

Different letter superscripts in the same row represent significant differences within groups, as tested by pairwise comparisons; presence of an ^c^represents a significant effect in the ANOVA

### Hypothesis 1. Individuals with eating disorders are more susceptible to both TSF and TAF than are healthy controls

#### Negative emotional responses to the TSF and TAF inductions: difficulty/distress composite on the target thought priming ratings scale

Normality was assessed by ensuring values for skewness were below 3 and kurtosis was below 10, following previous research [25]. All values fell under these cutoff scores. Violations of equality of covariance were also detected for this measure, which could not be corrected with transformations; however, given that ANOVA is robust to violations of this assumption [26] we proceeded with analyses. A significant effect of induction, as well as a group by induction interaction, was found for the difficulty/distress composite (see Table 1 for details). Paired t-tests were used to break down the interaction across groups, using a Bonferroni correction (*p* < .0167) to control for multiple comparisons within each group. For control participants, composite scores were significantly higher after the TAF induction than either the neutral or TSF induction. The difference between the TSF and neutral scores did not reach significance. For participants with eating disorders, composite scores were significantly different across all three inductions, all *p*’s ≤ .004. Independent sample t-tests (applying the Bonferroni correction) were conducted for across group comparisons, and the participants with eating disorders were demonstrated to have significantly higher levels of difficulty/distress than controls for the TSF induction (*t*(30.47) = 5.48, *p* < .001), but not for the TAF (*t*(42) = 0.398, *p* = .70), or neutral inductions (*t*(26.40) = 1.56, *p* = .136).

#### Emotional/behavioral responses (as measured by the State TSF scale)

Analysis on the emotional/behavioral subscale of the state TSF scale was performed to assess the effects of the inductions across groups. Equality of covariance was violated for this measure; however, because values for skewness and kurtosis were below cutoff values, we proceeded with analyses. A significant effect of induction (*F*_2,84_ = 25.14, *p* < .001, *η*_*p*_^*2*^ = .374) emerged, which was qualified by a significant group by induction interaction (*F*_2,84_ = 4.23, *p* = .018, *η*_*p*_^*2*^ = .092). Paired t-tests (using the Bonferroni correction) demonstrated that for the control group, TAF induction led to significantly higher scores than both neutral (*t*(22) = 4.87, *p* < .001) and TSF inductions (*t*(22) = 5.72, *p* < .001), with no significant differences emerging between the neutral and TSF induction. For participants with eating disorders, both the TSF induction (*t*(21) = 3.43, *p* < .005) and TAF induction (*t*(20) = 4.13, *p* < .001) led to higher scores than the neutral induction, with no significant difference between the TSF and TAF inductions. Independent t-tests (with Bonferroni correction) demonstrated that participants with eating disorders reported higher scores than controls for the neutral (*t*(31.09) = 4.79, *p* < .001), TAF (*t*(32.33) = 3.11, *p* < .005), and TSF (*t*(27.14) = 6.66, *p* < .001) inductions.

#### Body-related responses (as measured by the State TSF Scale)

**Fig. 1:**
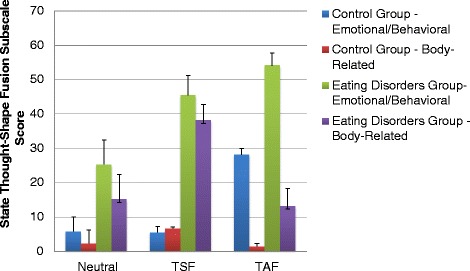
Scores on the subscales of the state Thought Shape Fusion (TSF) measure (i.e., emotional/behavioral subscale and body-related subscale, with standard error) across groups and induction condition (neutral, TSF, and Thought Action Fusion (TAF)

Analysis on the body-related subscale was also performed. Scores were subjected to a log transformation, to correct for violations of normality. Equality of covariance was violated for this measure; however, because values for skewness and kurtosis were below cutoff values, we proceeded with analyses. A significant effect of induction (*F*_2,84_ = 6.90, *p* < .005, *η*_*p*_^*2*^ = .141) emerged, but the group by induction interaction did not reach significance (*F*_2,84_ = 2.57, *p* = .083, *η*_*p*_^*2*^ = .058). Pairwise comparisons demonstrated that scores on the body-related composite score were significantly higher after the TSF induction than either the neutral or TAF inductions (*p* < .05). Figure 1 depicts the emotional/behavioral and body-related subscale raw scores across groups for each of the inductions.

#### Neutralization behavior

Significantly more participants with an eating disorder neutralized after receiving the TSF induction (47.6 %) than did controls (0 %), *χ*2 = 14.17 (*N* = 44, *df* = 1), *p* < .001. Similarly, more participants with eating disorders neutralized after receiving the TAF induction (61.9 %) than did controls (35 %), *χ*2 = 4.39 (*N* = 44, *df* = 1), *p* < .05. No participants in either group neutralized after the neutral induction.

### Hypothesis 2: Individuals with eating disorders will report more general susceptibility to TAF and Trait TSF than will healthy controls

#### TAF scale

Equality of covariance was violated for the TAF scale; however, because values for skewness and kurtosis were below cutoff values, we proceeded with analyses. Greenhouse-Geisser was used given violations of sphericity. No effect of induction emerged (*F*_1.61,82_ = 0.19, *p* = .78, *η*_*p*_^*2*^ = .005), nor was there a group by induction interaction (*F*_1.61,82_ = 0.117, *p* = .89, *η*_*p*_^*2*^ = .003). Participants with eating disorders reported significantly higher TAF scores averaged across the inductions (*M* = 32.83, *SD* = 14.30) than did control participants (*M* = 15.30, *SD* = 14.30), *F*_1,41_ = 16.06, *p* < .001, *η*_*p*_^*2*^ = .281.

#### Trait TSF scale

Equality of covariance was also violated for the trait TSF measure; however, because values for skewness and kurtosis were below cutoff values, we proceeded with analyses. No effect of induction emerged (*F*_2,80_ = 2.35, *p* = .10, *η*_*p*_^*2*^ = .055), nor was there a group by induction interaction (*F*_2,80_ = 0.79, *p* = .46, *η*_*p*_^*2*^ = .019). Participants with eating disorders reported significantly higher trait TSF scores averaged across the inductions than did control participants, *F*_1,40_ = 79.19, *p* < .001, *η*_*p*_^*2*^ = .643. The mean trait TSF score for participants with eating disorders across inductions was 25.55 (*SD* = 8.8), and for control participants was 2.27 (*SD* = 8.8)*.* Analyses on the total scores on the clinically-relevant trait TSF subscale demonstrated an identical pattern of results, such that no effect of induction emerged (*F*_2,84_ = 0.263, *p* = .77, *η*_*p*_^*2*^ = .006), nor was there a group by induction interaction (*F*_2,84_ = 0.825, *p* = .44, *η*_*p*_^*2*^ = .019). Between-subjects analyses (collapsed across inductions) demonstrated a main effect of group, such that participants with eating disorders had significantly higher clinically-relevant trait TSF scores than did control participants, *F*_1,42_ = 127.53, *p* < .001, *η*_*p*_^*2*^ = .752. The mean score for participants with eating disorders across the inductions was 11.46 (*SD* = 2.7), and for control participants was 2.20 (*SD* = 2.7).

## Discussion and conclusions

We predicted that individuals with eating disorders would be more susceptible to TAF and TSF inductions than would healthy controls (Hypothesis 1). The results supported Hypothesis 1, demonstrating that individuals with eating disorders had higher rates of neutralization behavior after TSF and TAF inductions than did healthy controls. Furthermore, participants with eating disorders reported more emotional distress (as measured by both the difficulty/distress composite and the emotional/behavioral subscale on the state TSF scale) after a TSF induction compared to controls. TAF was also successfully induced in both groups, with higher levels of difficulty/distress reported after the TAF induction as well as higher scores on the emotional/behavioral subscale of the state TSF measure, in comparison to the neutral induction in both groups. Of interest is the fact that the TAF induction was reported to be less vivid than the other inductions. Given the high level of distress reported by both groups of participants, it may be that they did not engage as deeply in the visualization of the TAF induction as a way of coping with the distressing image involved with the manipulation.

Hypothesis 2, which predicted that individuals with eating disorders would report more general susceptibility to TAF and TSF, was also supported. Individuals with eating disorders reported higher scores on both the trait TSF and TAF measure than did controls. Scores on these measures were stable across the inductions, suggesting that both tools are tapping into a relatively stable trait. Some conflicting evidence exists for the stability of the trait TSF measure. Some studies report that scores on this measure were stable regardless of manipulations of food-related thoughts [e.g., ([15]), whereas others have reported effects of TSF inductions on trait TSF scores [10, 17]. Recently, further validation of this measure demonstrated good test-retest reliability and stability of scores on this measure across experimental sessions [18], supporting the use of the trait TSF scale as a trait measure. The current findings support the distinction between state and trait TSF, and the use of the trait TSF measure for assessment of general propensity towards the experience of TSF.

Both groups (eating disorders and controls) reported higher levels of emotional/behavioral response (as measured by the state TSF scale) after the TAF induction, in comparison to a neutral induction. However, body-related composite scores were elevated after the TSF induction but not the TAF induction, with the interaction between group and induction condition failing to reach statistical significance. Therefore, the TAF induction appears to influence emotional/behavioral responses (i.e., anxiety, guilt, perceptions of wrong-doing, and urges to neutralize) in both controls and those with eating disorders. However, only the TSF induction was associated with changes in body-related perceptions (i.e., perceptions of weight gain and fatness). Some previous research has demonstrated that anxiety inductions can lead to perceptions of weight gain in chronic dieters [8]. This finding has been accounted for by the “body displacement” hypothesis, such that individuals with eating disorders or weight-related concerns may displace emotional distress onto their bodies [27, 28]. There is some preliminary evidence that body displacement is related to feelings of ineffectiveness in individuals with eating disorders [29]. However, the current findings do not suggest that body displacement is occurring after a TAF induction, given that there were no differences between scores on the body-related composite between the neutral and TAF inductions.

Although TAF and TSF have been primarily conceptualized as cognitive distortions, the metacognitive model of OCD [30] suggests that TAF is part of a metacognitive belief system known as “thought-fusion beliefs.” According to Wells [30], thought-fusion beliefs involve the perceived danger associated with thoughts. Recent research has demonstrated that metacognitive beliefs, including thought-fusion, are better predictors of treatment outcome in individuals with OCD than are obsessional thoughts about perfectionism and responsibility [31]. Grotte and colleagues [31] suggest that targeting metacognitive beliefs should be one of the primary aims of psychotherapy. It may be beneficial to consider TAF and TSF under the broader conceptualization of “thought-fusion” metacognitive beliefs, as opposed to studying them as distinct cognitive distortions. Both TSF and TAF may represent a general propensity towards obsessive thinking, rather than a phenomenon that is specific to eating disorders or OCD. TSF and TAF appear to be part of a global thinking style that is common in people who develop eating disorders--with TAF being just as characteristic as is TSF.

Interpretation of the current results is limited by the inclusion of a transdiagnostic sample of individuals with eating disorders. Some recent findings suggest potential differences in TSF across eating disorder subtypes in TSF [32], such that those with a diagnosis of AN-BP report higher levels of TSF than other groups. Future research would benefit from examining predictors of state TSF susceptibility across eating disorder subtypes. Further validation of the measures employed in the current study, especially the state TSF scale, is also needed. Although all measures employed in this study have been used in previous research, the psychometric properties of the state TSF scale and the Target Thought Priming Ratings measure have not yet been established. Given the final sample size of the study, we were also unable to employ full counterbalancing of the presentation of the inductions. All 6 potential orders of induction were employed, with 3–4 participants in each of the groups receiving each of the induction orders. However, we cannot fully rule out potential order effects without counterbalancing. A further potential limitation of the current methodology was the specific TAF induction that was employed. This induction as chosen based on previous research [19]. However, the fact that this induction involved asking participants to write that they “hope” that a loved one is in an accident induces a differential perception of moral wrong-doing than would merely asking them to imagine that a loved one is in an accident (e.g., [33]). The intentionality involved in the induction was somewhat different across the conditions, such that the TSF induction involved imagining eating a high-caloric food, but not being asked to write about “hoping” to eat a high-caloric food. These methodological nuances are not expected to have affected the general pattern of results, although further research on intentionality in TAF and TSF inductions is warranted.

This study is the first to examine the effects of TSF and TAF inductions in a sample of participants with eating disorders and healthy controls, using a within-subjects design. The high levels of TAF reported by the sample of individuals with eating disorders, combined with the high levels of neutralization behavior in participants with eating disorders, suggest the importance of further studying this construct. Clinically, these findings suggest that there may be a high propensity towards “thought-fusion” beliefs in individuals with eating disorders that are not only limited to food. Assessment of the obsessional thinking style, and presence of cognitive distortions, in clients with eating disorders, even in the absence of a diagnosis of OCD, may be informative in the development of patient-centered treatment plans. Further research on the clinical implications of the high levels of both TAF and TSF that are present in individuals with eating disorders is warranted.

## Footnotes

1 Two patients disclosed at follow-up that they had consulted a professional for psychological problems within the past three months. However, neither of these participants met criteria for any of the conditions assessed with the SCID-I-RV [21]. Given the absence of specific diagnoses relevant to this research, these participants were maintained in the analyses.

2 Participants completed questionnaires about eating pathology, obsessional thinking, depressive symptoms, as well as ego-dystonicity of eating-related thoughts. Additional details are available in Purcell Lalonde, O’Connor, Aardema & Coelho [34].
